# Improved functional *JAG1* and *NOTCH2* variant testing in patients with clinical or suspected Alagille syndrome using new low-Notch activity cells

**DOI:** 10.1007/s00439-026-02832-7

**Published:** 2026-04-18

**Authors:** Nicole Buhl, Eva-Doreen Pfister, Daniel V. Oliveira, Fabio Turetti, Eberhard Lurz, Ulrich Baumann, Nataliya Di Donato, Thomas Illig, Britta Skawran, Emma R. Andersson, Jan Mašek, Amelie Stalke

**Affiliations:** 1 Pediatric Gastroenterology and Hepatology, Hannover Medical School, Hannover, Germany; 2 Department of Cell Biology, Faculty of Science, Charles University, Viničná 7, Prague, 12800 Czech Republic; 3 Institute of Organic Chemistry and Biochemistry, Czech Academy of Sciences, Prague, Czech Republic; 4 Department of Pediatrics, Dr. von Hauner Children’s Hospital, University Hospital, LMU Munich, Munich, Germany; 5 Department of Human Genetics, Hannover Medical School, Carl- Neuberg-Str.1, 30625 Hannover, Germany; 6 Hannover Unified Bank, Hannover Medical School, Hannover, Germany; 7 Department of Cell and Molecular Biology, Karolinska Institute, Stockholm, Sweden

## Abstract

The autosomal dominant multisystemic Alagille Syndrome (ALGS) is an important cause of pediatric cholestasis. ALGS is associated with pathogenic variants in JAGGED1 (JAG1) or NOTCH2, ligand and receptor components of the Notch-signaling pathway, respectively. The detected missense variants are commonly classified as variants of uncertain significance, hindering ALGS diagnosis. To overcome this issue, we have developed a CRISPR/Cas9-engineered Low-Notch activity (LNA) cells allowing for selective testing of JAG1-NOTCH2 signaling activity. We tested this approach on 9 patients with pediatric hepatopathies with phenotypes ranging from the full clinical ALGS spectrum to isolated neonatal cholestasis and atypical ALGS abnormalities who carried 5 *JAG1* and 3 *NOTCH2* missense variants of interest. Additionally, western blot analyses revealed an effect on protein expression for two *JAG1* missense variants, one of which had altered glycosylation, potentially indicating pathogenic effects. For this *JAG1* and one *NOTCH2 de novo* missense variant, luciferase-based Notch reporter activity was significantly reduced in LNA cells, while no change was observed in commonly used HEK293T, Huh7, or Hep2G cells. These findings allow independent confirmation of recently classified c.53T>G p.(Leu18Arg) in *JAG1*, and a re-classification of c.1235G>T p.(Cys412Phe) in *NOTCH2* as likely pathogenic based on ACMG criteria. Collectively, we provide evidence that selective testing of JAG1-NOTCH2 interaction in the newly developed CRISPR-engineered cells, combined with a glycosylation assay, enables robust functional evaluation of ALGS-associated *JAG1* and *NOTCH2* variants.

**Supplementary Information:**

The online version contains supplementary material available at 10.1007/s00439-026-02832-7.

## Introduction

Alagille Syndrome (ALGS) is a multisystemic autosomal dominant disorder predominantly affecting the liver, heart, face, skeleton, kidneys, vasculature, and eyes. The wide clinical spectrum ranges from life-threatening liver or heart disease to merely subclinical manifestations, including butterfly vertebrae, posterior embryotoxon, pulmonary artery stenosis, and characteristic facial features. One of the most clinically severe aspects of ALGS stems from disturbed biliary tree development during embryogenesis, making it a common cause of hereditary cholestasis in children (Alagille et al. 1987; Balistreri and Bezerra 2006; Spinner et al. 1993). Most cases carry pathogenic variants in *JAG1* (~ 94%; MIM#118450), encoding the Notch signaling pathway ligand JAG1. In contrast, far fewer pathogenic variants are known for the NOTCH2 receptor-encoding *NOTCH2* gene (MIM#610205), although numerous variants of uncertain significance (VUS) have been reported (ClinVar 2025; Fokkema et al. 2021; Landrum et al. 2014; LOVD 2025).

Notch signaling regulates cell fate determination during development and maintains adult tissue homeostasis. The interaction of Notch receptors with their ligands via direct cell-to-cell contact depends heavily on post-translational glycosylation of both ligands and receptors, affecting the Jag1-Notch2 interaction during bile duct development (Niknejad et al. 2023; Urata and Takeuchi 2020). Under physiological conditions, JAG1, expressed by the liver periportal mesenchyme (Mašek and Andersson 2024), activates the NOTCH2 receptor (Geisler et al. 2008), expressed by differentiating cholangiocytes within the surrounding ductal plate, driving their differentiation and bile duct lumenization (Hofmann et al. 2010). JAG1-NOTCH2 interaction triggers proteolytic NOTCH2 cleavage and release of the receptor’s intracellular NOTCH domain (NICD) (Mašek and Andersson 2017; Penton et al. 2012). The NICD then translocates to the nucleus, where it interacts with RBPJk, a DNA-binding protein of the CBF1/Suppressor of Hairless/LAG-1 (CSL) family, and its co-activator, MAML, to activate Notch target gene transcription. When genetic variants disrupt the JAG1/ NOTCH2 signaling axis, ALGS may occur, depending on the nature of the respective variant.

Overall, complications associated with and arising from bile duct developmental defects contribute to disease progression and fibrosis, ultimately resulting in liver transplantation or death in over 60% of affected patients (Mašek et al. 2024; Vandriel et al. 2023). ALGS-typical phenotypic manifestations may not be present or evident in all patients due to incomplete penetrance and variable expressivity, and a clear classification of, especially missense variants in the two ALGS-associated genes *JAG1* and *NOTCH2*, is gaining increasing attention to establish a diagnosis. Easily accessible primary patient-derived materials, such as whole blood, lymphoblastoid cell lines, or fibroblasts, are of limited use for functional analyses as *JAG1* is expressed only at low levels, and together with other Notch ligands and receptors, does not allow for direct assessment of JAG1-NOTCH2 signalling, relevant for ALGS.

In this study, we present a novel in vitro system for quantifying and defining the impact of variants on JAG1-NOTCH2 activity. Using this approach, we describe 5 *JAG1* missense and 3 *NOTCH2* missense variants of interest detected in 9 patients with suspected ALGS and investigated the functional consequences to assess the pathogenicity and the underlying pathomechanism of these variants.

## Materials and methods

For details, see Supplementary Methods and Results.

### Patient samples

All patients exhibited a liver phenotype with neonatal cholestasis with elevated gamma-glutamyl transferase (GGT) levels as the primary manifestation within the first three months of life. Nine patients underwent diagnostic work-up at the Pediatric Gastroenterology and Hepatology department of Hannover Medical School, and one patient at the Department of Pediatrics of University Hospital in Munich. Patients were identified by genetic analyses on blood samples to have VUS in *JAG1* or *NOTCH2*. The study protocol conforms to the ethical guidelines of the Declaration of Helsinki (1964) and has been approved by the ethics committee of Hannover Medical School (No 2591 − 2015 and No 7656 − 2017). The patients and/or parents gave written informed consent.

### HEK 293T low Notch activity (HEK293T-LNA) cells generation by CRISPR/Cas9 mutagenesis and cell culture

Human embryonic kidney cells HEK293T (kindly provided by Johann Meyer, Institute of Experimental Hematology, MHH, Hannover, Germany) were cultured in Dulbecco’s Modified Eagle Medium (DMEM, Merck, #D5796), 10% fetal calf serum (FCS, Merck, #S0615), 1% sodium pyruvate (Merck, #S8636) and 100 U/mL penicillin/streptomycin (sigma, #P0781). Human liver carcinoma cell lines HepG2 and Huh7 (kindly provided by Professor Nam-Ho Huh, Okayama University, Japan) were cultured in DMEM (PAN-Biotech, #P04-05550) containing 10% FCS and 100 U/mL penicillin/streptomycin.

We used the *NOTCH1/2/3* KO HEK293T (Kindly provided by Urban Lendahl, Karolinska Institute, Sweden) (Wu et al. 2021) to develop a new HEK293T cell line, depleted of NOTCH1/2/3 receptors and multiple Notch ligands, and thus largely devoid of Notch signaling activity – Low Notch Activity HEK293T cells (HEK293T-LNA cells). We used the CRISPR/Cas9 px459 system (AddGene #62988) (Cong et al. 2013), performing multiple rounds of sgRNA/Cas9 transfection and selection, followed by limiting dilution and clonal expansion. The clones from individual cells were tested by Western blotting, qPCR, and sequencing (Bell et al. 2014). Resulting HEK293T-LNA cells are *NOTCH1/2/3*^*−/−*^, *JAG1/2*^*−/−*^, *DLL1*^*−/in frame del*^, *DLL3*^*−/in frame del*^, *DLL4*^*+/−*^ (Fig. S3A–D, G). JAG1-T2A-EGFP-expressing HEK293T-LNA cells originate from HEK293T-LNA cells with random genomic integration of the transiently transfected human *JAG1-T2A-EGFP* plasmid that were purified by FACS. Western blotting confirmed JAG1 over-expression (Fig. S3E). Both HEK293T-LNA and HEK293T JAG1-T2A-EGFP HEK293T-LNA cells were cultured in DMEM (High glucose, Glutamax supplement, pyruvate, Thermo Fisher #31966047), supplemented with 10% fetal bovine serum (FBS, Gibco™, #A5256701), and 1% of penicilin/streptomycin (Gibco™, #15140122). All cell lines were cultivated at 37 °C and 5% CO_2_ in a humidified incubator.

### Vector constructs

For reporter activity assays with pcDNA3_*JAG1* or pcDNA3.1_*JAG1* and pcDNA3.1_*NOTCH2* expression vectors containing the cDNA of *JAG1* (NM_000214.3) and *NOTCH2* (NM_024408.4), were purchased from Genescript in the pcDNA3.1 backbone. The *JAG1-EGFP* vector was subsequently modified using EcoRI and NotI sites to contain a self-cleaving T2A peptide between JAG1 and EGFP, resulting in *JAG1-T2A-EGFP* in pcDNA3.1.

For CRISPR/Cas9 mutagenesis, the px459 vector (AddGene #62988) was used. The gRNA target sites were selected with the CHOP CHOP tool (Labun et al. 2019) (Fig. S3F).

All *NOTCH2* and *JAG1* variants were generated using QuickChange Lightning Site-Directed Mutagenesis Kit (Agilent Technologies, 210518) according to the manufacturer’s protocol. For primer sequences, see Table S1. All sequences were verified by Sanger sequencing either for the insert after cloning into a new backbone or by Full PlasmidSeq (microsynth SeqLab, Göttingen, Germany). As a plasmid reporter, Ga981-6 with 12 CSL binding sites upstream of the firefly luciferase open reading frame (12x CSL) (Kato et al. 1997) was used (Kind gift by Urban Lendahl, Karolinska Institute, Sweden).

### Transfection

To overexpress JAG1, NOTCH2, or their mutant variants for western blot, 325,000 HEK293T, 500,000 Huh7, or 550,000 HepG2 cells were seeded per well 24 h before transfection in a 6-well format. For HEK293T or Huh7 transfection, pcDNA3 or pcDNA3.1 vector, Lipofectamine 2000 (Thermo Fisher, #11668019), and MIGR1 (kindly provided by Warren Pear via Addgene #27490) were used. For HepG2 transfection, the respective pcDNA3.1 vector, Lipofectamine 3000 (Thermo Fisher, L3000008) were used. The empty vector served as the negative control. The medium was changed after 24 h. Whole-cell lysates were collected 48 h post-transfection.

### Western blot

Harvested cells were lysed in RIPA buffer containing protease and phosphatase inhibitors (Thermo Scientific™, #A32961). The influence of JAG1 variants on protein glycosylation was analyzed by digestion with Peptide-N-glycosidase F (PNGase F) (New England Biolabs, #P0704S). Proteins were separated on an SDS-PAGE and transferred onto a PVDF or nitrocellulose membrane. Membranes were blocked for 1 h in 5% non-fat dry milk in TBS-T at room temperature. After incubation with primary rabbit-anti JAG1 (Cell Signaling, #70109S, 1:1000) or rabbit-anti NOTCH2 antibody (Cell Signaling, #5732S, 1:1000-1:3000) (for further antibodies used, see Table S2) overnight at 4 °C and secondary antibodies for 1 h at room temperature, the membrane was developed using ECL substrate.

### Luciferase-based Notch signaling co-culture assays

For HepG2/Huh7 assays, mycoplasma-free cells (20,000/well) were seeded in white 96-well plates. After 24 h, signal-receiving cells were transfected (Lipofectamine 2000/3000, Thermo Fisher, ##11668019, L3000008) with 40 ng 12xCSL-luc, 6 ng (Huh7) or 3 ng (HepG2) pGL4.70 (Promega #E6881; Renilla), and 50 ng pcDNA3.1_*NOTCH2* or empty vector. Signal-sending cells were transfected with 50 ng pcDNA3.1_*JAG1*_WT or empty vector. After 6 h, signal-sending cells were detached (TrypLE, Thermo Fisher #12563029) and co-cultured with signal-receiving cells. Luciferase activity was measured at 48 h using the Dual-Glo system (Promega #E2920).

For HEK293T-LNA cell assays, cells (1.25 × 10⁵/well in 24-well plates) were transfected (Lipofectamine 2000, #11668019) with 200 ng 12xCSL-luc, 8 ng Renilla, and 100 ng either *NOTCH2* (wild-type/variant) or empty pcDNA3.1 plasmid (DNA was adjusted to 1 µg by adding pBluescript KSII, kind gift of Zbynek Kozmik, IMG Prague, Czechia). The next day, 10,000 transfected signal-receiving cells were co-cultured with 40,000 JAG1-expressing signal-sending cells in 96-well plates. Luciferase activity was quantified at 40 h post-transfection (Dual-Glo, Promega #E2940). For *JAG1* variants, signal-sending cells were transfected with *JAG1* (wild-type/variant) or empty vector, then co-cultured with *NOTCH2*-transfected signal-receiving cells. Activity was measured as above.

Raw data of the luciferase assays can be found here: https://www.ebi.ac.uk/biostudies/studies/S-BSST2221.

### Sequencing approach

DNA was extracted from whole blood samples. Exome sequencing libraries were prepared using the xGen Exome Research Panel (Integrated DNA Technologies, Inc., Coralville) or TruSeq Exome (Illumina, San Diego, CA) and sequenced on a NextSeq 500/550 or MGI DNB SEQ-G400RS sequencer. Patient 3 was retrospectively analyzed and had prior external *JAG1* testing via single-strand conformation polymorphism analysis and cycle sequencing. Further genetic analyses were not possible as this patient was lost to follow-up.

Variant nomenclature refers to NCBI transcripts *JAG1* (NM_000214.3) and *NOTCH2* (NM_024408.4).

### Variant classification

The variants were classified according to the American College of Medical Genetics and Genomics (ACMG) Standards and Guidelines using the point system and the ClinGen Variant Classification Guidance (ClinGen 2025; Richards et al. 2015; Tavtigian et al. 2020).

## Results and discussion

### Case reports

All patients of our cohort showed neonatal cholestasis with elevated GGT levels as the first manifestation within the first three months of life and carried a heterozygous missense variant in *JAG1* or *NOTCH2* (Tables 1 and 2). All *JAG1* and *NOTCH2* variants, detected in these patients and classified as VUS in the beginning of the study, were included.

**Table 1:** Overview of data from patients carrying a *JAG1* missense variant

Patient		1	2	3	4	5
Sex		m	m	f	m	m
ALGS-characteristic	Liver	Neonatal cholestasis, biliary cirrhosis	Neonatal cholestasis, biliary cirrhosis	Neonatal cholestasis	Neonatal cholestasis, biliary cirrhosis	Only transient neonatal cholestasis
	Histology	At 5 years: complete cirrhosis (F6) with severe ductopenia	At 2 months: neonatal hepatitis, fibrosis (F2)	At 1 month: periductal inflammation, portal fibrosis (F2-3)	At 1 month: severe ductopenia, fibrosis (F2)	Not conducted
	Heart	Pulmonary stenosis	No	Pulmonary stenosis	Pulmonary stenosis	No
	Face	Yes	No	Yes	Yes	No
	Kidneys	No	No	No	Renal cysts, impaired renal function	No
	Skeleton	Butterfly vertebrae	Ulna minus variant	Butterfly vertebrae	No	Unknown
	Vasculature	Development of moyamoya syndrome during teenage years	No	No	No	No
	Eyes	Embryotoxon	No	No	No	Unknown
SGA		Yes	No	Yes	Yes	No
Clinical ALGS		Yes	No	Yes	Yes	No
LTx		Yes (5 years)	Yes (1 year)	Unknown, no follow-up	Death (on waiting list due to decompensated liver cirrhosis)	No (alive)
Gene		NM_000214.3 (*JAG1*)				
Variant		c.53T>G	c.316A>G	c.1345A>G	c.1912T>C	c.3467T>C
		p.(Leu18Arg)	p.(Thr106Ala)	p.(Ile449Val)	p.(Cys638Arg)	p.(Val1156Ala)
Zygosity		Heterozygous	Heterozygous	Heterozygous	Heterozygous	Heterozygous
Inheritance		Unknown	Unknown	Unknown	Unknown	Unknown

*ALGS* Alagille syndrome, *f* female, *LTx* liver transplantation, *m* male, *SGA* small for gestational age

**Table 2:** Overview of data from patients carrying a *NOTCH2* missense variant

Patient		6	7	8	9
Sex		f	f	m	f
ALGS-characteristic	Liver	Neonatal cholestasis	Neonatal cholestasis, liver fibrosis	Neonatal cholestasis	Neonatal cholestasis, liver cirrhosis
	Histology	Not conducted	Biopsy at 3 months: minimal lobular and portal inflammation, portal fibrosis (F2-3)	ERCP at 2 months with hypoplastic bile ducts, biopsy at 9 years: portal fibrosis (F3-4), ductular reaction	Biopsy at 1 month: Bile duct proliferates, advanced fibrosis (F4-5)
	Heart	No	Atrial septal defect type 2	Bicuspid aortic valve	No
	Face	No	No	Yes	No
	Kidneys	No	No	No	No
	Skeleton	No	No	No	Vertebral anomaly
	Vasculature	No	No	No	Vascular anomalies including azygos continuation, absence of inferior vena cava and vena portae with separate inlets of vena lienalis and vena mesenterica
	Eyes	No	No	No	No
Additional remarks				Growth retardation	Jejunal atresia, in summary several signs of the heterotaxy syndrome
SGA		Yes	Yes	Yes	No
Clinical ALGS		No	No	Yes	No
LTx		No (alive)	No (alive)	No (alive)	Yes (7 months)
Gene		NM_024408.4 (*NOTCH2*)			
Variant		c.1235G>T	c.3995G>A	c.6094C>A	c.6094C>A
		p.(Cys412Phe)	p.(Arg1332His)	p.(His2032Asn)	p.(His2032Asn)
Zygosity		Heterozygous	Heterozygous	Heterozygous	Heterozygous
Inheritance		de novo	Unknown	Paternal	Unknown

Patients 7–9 have previously been reported by Stalke et al. (Stalke et al. 2018)

*ALGS* Alagille syndrome, *f* female, *LTx* liver transplantation, *m* male, *SGA* small for gestational age

Patient 1 (male, *JAG1*: c.53T>G p.(Leu18Arg): he was a small for gestational age (SGA) newborn and showed, besides neonatal cholestasis and embryotoxon, also ALGS-typical facial features, cardiac and skeletal anomalies, including pulmonary artery stenosis and butterfly vertebrae. The patient thus presented a classic clinical case of ALGS as defined by the existence of at least 3 of the 7 major clinical features (hepatic, cardiac, facial, ocular, skeletal, renal, vascular) (Ayoub and Kamath 2020). The patient underwent a successful liver transplantation due to biliary cirrhosis (based on histological assessment) at the age of five years. During his teenage years, he developed moyamoya syndrome.

Patient 2 (male, *JAG1*: c.316A>G p.(Thr106Ala)): he showed mild skeletal abnormalities e.g. ulna minus variant. He developed a rapidly progressive liver cirrhosis and had a successful liver transplant at the age of one year.

Patient 3 (female, *JAG1*: c.1345A>G p.(Ile449Val)): she presented a classic clinical case of ALGS. She was an SGA newborn and had a neonatal cholestasis, pulmonary artery stenosis, facial anomalies, and butterfly vertebrae. Due to loss of follow-up, further data, such as the patient’s transplant status, are not known.

Patient 4 (male, *JAG1*: c.1912T>C p.(Cys638Arg)): he presented with pulmonary stenosis, typical facial features, renal cysts, and impaired renal function in addition to neonatal cholestasis with subsequent development of biliary cirrhosis. Overall, the patient showed the typical clinical signs of ALGS. The patient died due to decompensated liver cirrhosis before transplantation.

Patient 5 (male, *JAG1*: c.3467T>C p.(Val1156Ala)): he showed only transient neonatal cholestasis without further ALGS symptoms.

Patient 6 (female, *NOTCH2*: c.1235G>T p.(Cys412Phe), *de novo*), Fig. S1): she was a SGA newborn and presented with neonatal cholestasis and failure to thrive.

Patient 7 (female, *NOTCH2*: c.3995G>A p.(Arg1332His)): she showed only hepatic fibrosis and atrial septal defect type 2 with no further typical ALGS symptoms. She has not yet undergone a liver transplantation.

Patient 8 (male) and patient 9 (female) (*NOTCH2*: c.6094C>A p.(His2032Asn)): patient 8 showed a classic clinical case of ALGS due to neonatal cholestasis, growth retardation, a bicuspid aortic valve, and facial anomalies. However, patient 9, who also carries the variant c.6094C>A, showed neonatal cholestasis, jejunal atresia, and vascular anomalies, including azygos continuation, absence of inferior vena cava and vena portae with separate inlets of vena lienalis and vena mesenterica. In summary, she has several signs of the heterotaxy syndrome and not the typical ALGS signs. She underwent successful liver transplantation at seven months of age due to liver cirrhosis.

The assessment of missense variants in *JAG1* and *NOTCH2* is often challenging, partly due to the variable expressivity and incomplete penetrance of ALGS. Functional analyses can assist in this regard; however, readily accessible patient material is not particularly suitable for this purpose. We therefore used different cell-line-based in vitro approaches to characterize 5 *JAG1* and 3 *NOTCH2* missense variants of interest detected in the above-described 9 patients with suspected or clinical ALGS. Here, we demonstrate, particularly for *JAG1* variants, that variant-specific influences on the Notch signaling pathway might be masked by baseline Notch activity of the WT cell lines. By using our newly generated HEK293T-low-Notch activity (LNA) cell line, we were able to overcome this problem, making it a valuable tool for more sensitive and targeted variant analysis of Notch pathway components.

### Luciferase-based Notch signaling assay using standard cell lines failed to reveal an impact of *JAG1* variants on Notch signaling activity

**Fig. 1:**
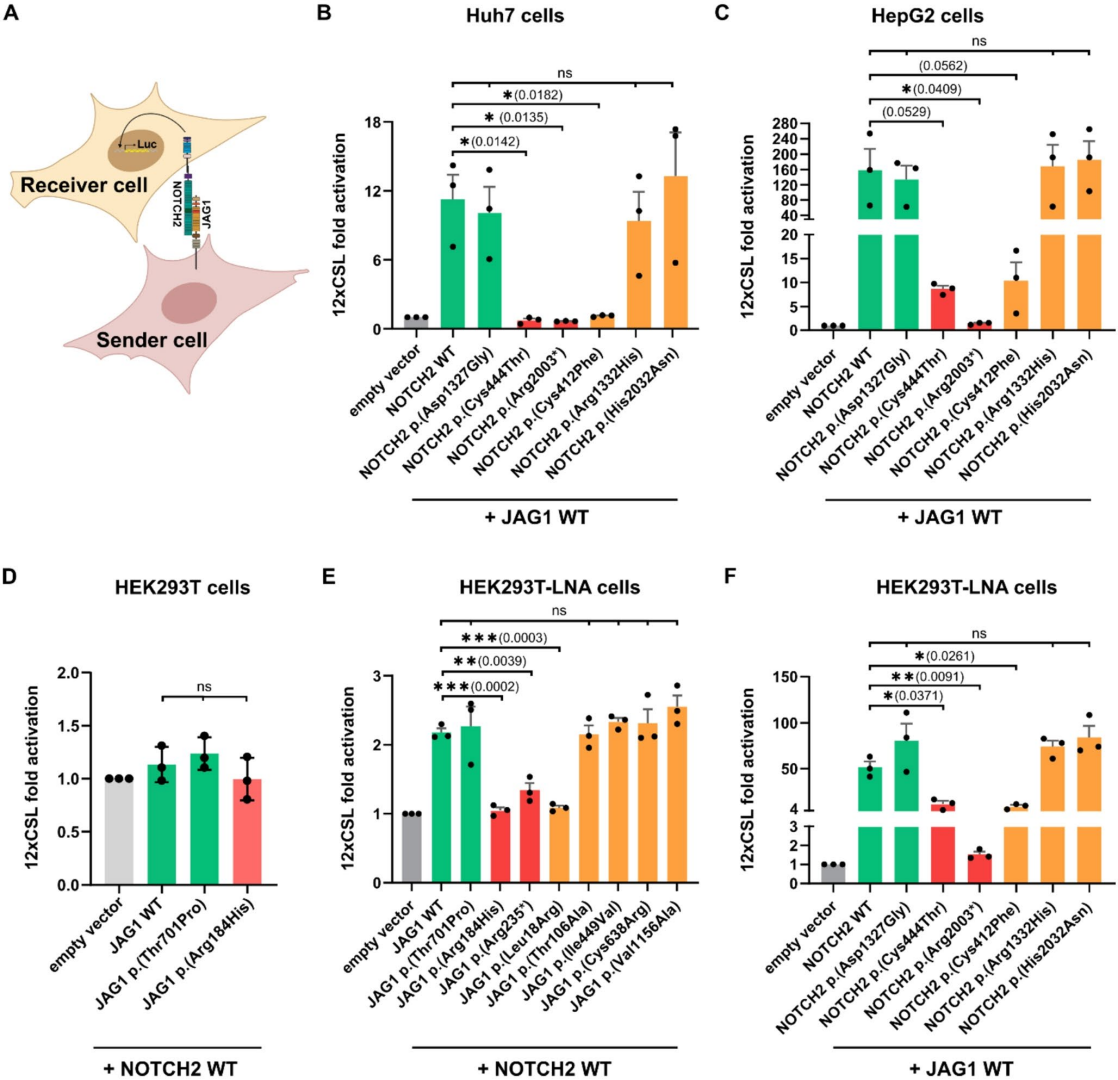
Effect of different JAG1 and NOTCH2 variants on the Notch signaling pathway activity. **A** Schematic representation of Notch signaling activity reporter co-culture setup. The JAG1 extracellular domain binds to the EGF repeats of NOTCH2. This leads to sequential cleavage of NOTCH2, releasing the Notch intracellular domain (NICD). NICD translocates to the nucleus, and activates the transcription of downstream target genes. This activation was measured using a firefly luciferase reporter system driven by 12xCSL binding sequence and normalized to Renilla luciferase activity. Luciferase activity was normalized to the empty vector control. **B**, **C** 12xCSL luciferase fold activation in co-cultures of Huh7 (**B**) or HepG2 (**C**) cells transiently expressing different NOTCH2 receptor variants (receiver cells) and Huh7 (**B**) or HepG2 (**C**) cells transiently expressing JAG1 WT (sending cells). **D**–**F** 12xCSL luciferase fold activation in co-cultures of **D** WT HEK293T transiently expressing NOTCH2 WT (receiver cells) and WT HEK293T cells transiently expressing different JAG1 variants (sending cells), **E** HEK293T-LNA cells transiently expressing NOTCH2 WT (receiver cells) and HEK293T-LNA cells transiently expressing different JAG1 variants (sending cells), or **F** HEK293T-LNA cells transiently expressing different NOTCH2 variants (receiver cells) and HEK293T-LNA cells stably expressing JAG1 WT (sending cells). Statistical significance was assessed using an ordinary one-way ANOVA with Dunnett’s multiple comparisons test. * *P* ≤ 0.05; ** *P* ≤ 0.01; *** *P* ≤ 0.001; **** *P* ≤ 0.0001, *ns* not significant. Error bars indicate standard error of the mean (SEM). Data are presented from *n* = 3 biological replicates, each with at least *n* = 2 technical replicates. Variants of interest are shown in orange, pathogenic controls in red and wildtype (WT) and benign controls in green

To test the effects of *NOTCH2* and *JAG1* variants on Notch signaling activity, we used the luciferase-based Notch signaling reporter assay (Fig. 1A) in human liver Huh7 and HepG2 cell lines, as well as human embryonic kidney cell line HEK293T. As pathogenic missense control, we used the two known ALGS-associated variants *JAG1* c.551G>A p.(Arg184His) and *NOTCH2* c.1331G>A p.(Cys444Tyr), both reported to result in functional impairment (Bell et al. 2014; Labun et al. 2019). As further pathogenic controls, we used the known ALGS-associated nonsense variants *JAG1* c.703C>T p.(Arg235*) and *NOTCH2* c.6007C>T p.(Arg2003*) which are expected to result in truncated proteins in our expression system. As benign controls, we used the common variants *JAG1* c.2101A>C p.(Thr701Pro) and *NOTCH2* c.3980A>G p.(Asp1327Gly) (minor allele frequency in gnomAD subpopulations: 5%). See also Tables S3 and S4 for details on control variants. For better readability and because we primarily consider effects at the protein level, we now refer to the variants only by their p. and not c. nomenclature.

While the benign NOTCH2 control p.(Asp1327Gly) (green) showed no significant difference compared to the WT, the pathogenic NOTCH2 controls p.(Cys444Tyr) and p.(Arg2003*) (red) showed, as expected, a significant reduction in signaling activity in Huh7 and HepG2 (Fig. 1B, C). However, no significant difference in reporter activity was observed for the WT JAG1, the benign control p.(Thr701Pro), nor the pathogenic control p.(Arg184His), in these two cell lines (Fig. S2A, B) or in the WT HEK293T cell line (Fig. 1D).

### Luciferase-based Notch signaling assay using a new HEK293T low-Notch activity cell line (HEK293T-LNA) revealed an impact of *NOTCH2* as well as *JAG1* variants on Notch signaling activity

Huh7, HepG2, or HEK293T are human cancer or immortalized cell lines, respectively. We hypothesised that the expression of multiple NOTCH receptors and ligands, including high levels of the endogenous “WT” JAG1 (Kuintzle et al. 2024) could obscure the measurement of the effects of tested variants (Giovannini et al. 2006; Kunnimalaiyaan et al. 2015). Therefore, we developed a new cell line devoid of interference from the majority of the endogenously expressed Notch signaling components using CRISPR/Cas9 (Cong et al. 2013). The resulting HEK293T Low Notch Activity *NOTCH1/2/3*^*−/−*^, *JAG1/2*^*−/−*^, *DLL3*^*−/in frame del*^, *DLL1*^*−/in frame del*^, *DLL4*^*+/−*^ cells (HEK293T-LNA) have negligible Notch pathway activity as tested by the Notch reporter assay and loss of endogenous expression of Notch target HES1 (Fig. S3A–D). The HEK293T-LNA cells thus allowed us to specifically test the signaling potential of the respective *JAG1-NOTCH2* variants in a low-Notch activity context. We co-cultured signal-receiving HEK293T-LNA cells transiently expressing NOTCH2 variants together with signal-sending HEK293T-LNA cells stably expressing WT JAG1-T2A-EGFP (Fig. 1F, S3E) or signal-sending HEK293T-LNA cells transiently expressing JAG1 variants together with signal-receiving HEK293T-LNA cells transiently expressing WT NOTCH2 (Fig. 1E).

As expected, the co-culture of WT receptor and ligand-expressing cells led to a robust Notch reporter activation. Failure to elicit Notch signaling activity could be readily observed for pathogenic NOTCH2 (p.(Cys444Thr), and p.(Arg2003*), in red), and JAG1 (p.(Arg184His), and p.(Arg235*), in red) control variants (Fig. 1E, F). Among the variants of interest, JAG1 p.(Leu18Arg) and NOTCH2 p.(Cys412Phe) led to a significant reduction in luciferase activity. In contrast, the four JAG1 variants p.(Thr106Ala), p.(Ile449Val), p.(Cys638Arg) and p.(Val1156Ala) and the two NOTCH2 variants p.(Arg1332His) and p.(His2032Asn) (orange) showed no differences compared to the WT (Fig. 1B, C, E, F).

Interestingly, the overexpression of JAG1 p.(Leu18Arg) led to only a mild protein increase compared to the empty vector, suggesting low stability of the variant, and likely explaining the lower activation (Fig. S3G). The NOTCH2 p.(Cys412Phe) variant with reporter activity reduced by 77.3% compared to the WT, was expressed to the same extent as other tested NOTCH2 variants (Fig. S3H), suggesting impaired signaling capacity rather than protein stability defect.

Based on our functional data, the NOTCH2 variant p.(Cys412Phe) can be reclassified from VUS to likely pathogenic according to the ACMG criteria (Table S4). The variant is localized in the 10th of the 36 NOTCH2 ligand-binding EGF-like repeats. Cysteines in EGF-like repeats, as present here in the WT, have already been described for many other proteins, including JAG1, as crucial for correct protein folding (Gilbert et al. 2019; Le Caignec et al. 2002).

Interestingly, the SGA patient carrying p.(Cys412Phe) initially presented with neonatal cholestasis but showed no other ALGS-typical features (Table 2). The patient is currently clinically asymptomatic with a mild liver phenotype (elevated bile salt concentration, alkaline phosphatase activity under ursodeoxycholic acid therapy), underlining the highly variable expressivity in ALGS. The reasons for variable expressivity may lie in epigenetic processes and the influence of modifier genes that activate rescue mechanisms. A possible rescue mechanism might be described by Schaub et al., who showed that hepatocytes can transdifferentiate into mature cholangiocytes via a Notch-independent, TGF-beta-driven pathway in mice (Schaub et al. 2018). If true also in humans, it might explain why functional deficits detected in vitro do not predict severe clinical outcomes in every patient.

### Protein glycosylation assay can identify JAG1 variants with disrupted post-translational processing

**Fig. 2:**
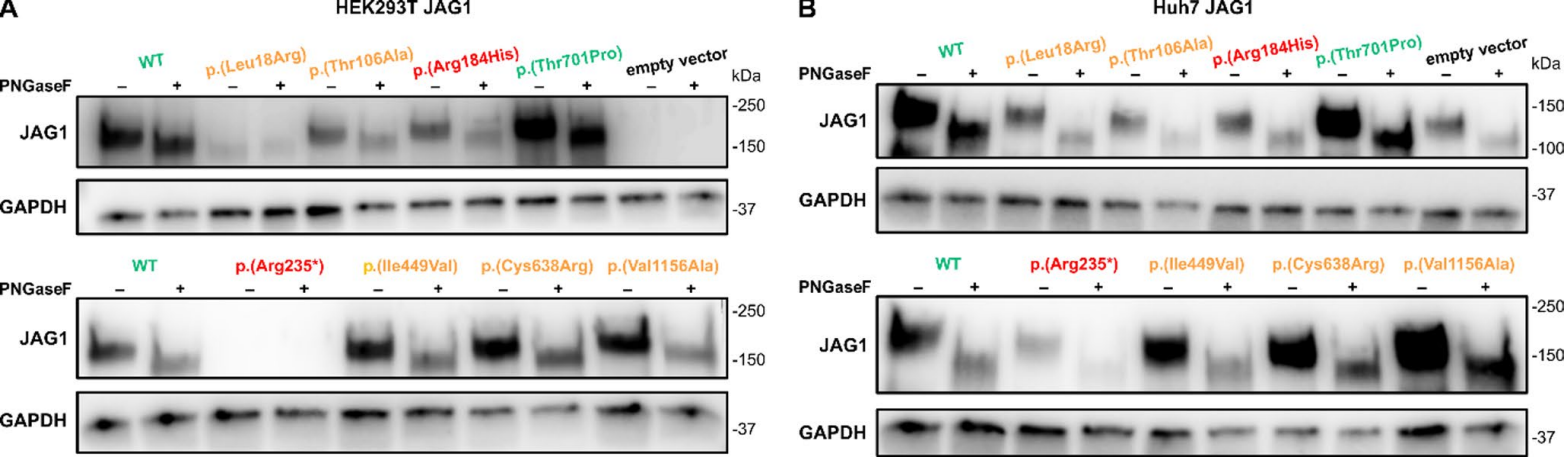
Effect of *JAG1* variants on protein levels by Western blot and additional PNGase F digestion. JAG1 was overexpressed in both **A** HEK293T and **B** Huh7 cells. Endogenous JAG1 expression was visualized by the empty vector. GAPDH was used as a loading control. Variants of interest are shown in orange, pathogenic controls in red, and wild-type (WT) and benign controls in green

Next, we expanded our western blot analysis from HEK293T-LNA cells (Fig. S3G) to HEK293T and Huh7 cells, which have endogenous expression of Notch components, to assess JAG1 variants protein expression also in the “WT” environment (Fig. 2A, B). In all cell lines, the protein levels of tested JAG1 variants (p.(Leu18Arg), p.(Thr106Ala), orange) were lower than those from WT and benign p.(Thr701Pro) JAG1-expressing control (green), despite the identical transfection conditions. The two JAG1 pathogenic controls (p.(Arg184His) and p.(Arg235*), red), showed also a visible reduction. Of note, the C-terminally truncated control variant p.(Arg235*) was not detected by the antibody directed against a C-terminal epitope and thus only endogenous signals were detected in both cell lines. Interestingly, in Huh7, only endogenous and no mutant JAG1 signal was detectable for the two variants p.(Leu18Arg) and p.(Thr106Ala), highlighting the usefulness of HEK293T-LNA cells (Fig. 2B). For p.(Leu18Arg), a lower band was detected in HEK293T compared to WT, which indicates a protein with a lower molecular weight (Fig. 2A).

JAG1 glycosylation was shown to be critical for its localization, since impaired glycosylation can cause protein accumulation in the endoplasmic reticulum (ER) (Meng et al. 2022; Sullivan et al. 2020), and work from Niknejad and colleagues recently demonstrated that Jag1 haploinsufficiency can be rescued by modulating the Jag1 and Notch2 glycosylation in mice (Niknejad et al. 2023), highlighting its role in ALGS pathology.

To test if altered glycosylation contributes to the p.(Leu18Arg) reduced activity, PNGase F digestion was performed on proteins isolated from HEK293T and Huh7 cell lines. The digestion removed all N-linked oligosaccharides and reduced the molecular weight of all analyzed proteins to the levels of undigested p.(Leu18Arg) in HEK293T cells, indicating this variant is not glycosylated. As a recent high-throughput study already demonstrated that this variant leads to reduced membrane expression levels (Gilbert et al. 2024), allowing for the reclassification of this variant as likely pathogenic, our experiment serves as an independent validation of the aforementioned high-throughput approach.

A plausible explanation for the abnormal membrane expression is that, since the mutation targets the JAG1 signal peptide, it disrupts the co-translational protein transport to the ER (Gutierrez Guarnizo et al. 2023). This could activate degrading quality control pathways such as “regulation of aberrant protein production” (RAPP) (Karamyshev et al. 2014; Karamysheva and Karamyshev 2023; Pinarbasi et al. 2018; Tikhonova et al. 2019, 2022), consistent with low protein levels detected by us (Fig. 2 and Fig. S3F) (for further information of the hypothetical mechanism see Fig. S4).

The expectable impairment of Notch signaling due to mislocalization and reduced expression levels of JAG1, was also reflected in significantly reduced Notch reporter activity in the HEK293T-LNA cells for p.(Leu18Arg) (Fig. 1E), in line with the severity of patient 1 clinical ALGS phenotype (Table 1).

In summary, according to the ACMG standards and guidelines (ClinGen 2025; Richards et al. 2015; Tavtigian et al. 2020), we independently validated the recent classification of variant of interest JAG1 p.(Leu18Arg), and provided evidence for re-classification of NOTCH2 p.(Cys412Phe) as likely pathogenic (see Tables S3 and S4 for variant characteristics, classification before and after this study, and applied ACMG criteria).

### Limitations of the study and future avenues

As we are introducing new tools for functional assessment of ligands’ and receptors’ VUS involved in Notch-driven genetic disorders, we next discuss their limitations and future applications. First, our functional analyses cannot capture all pathogenic effects. Variants with no alteration of Notch signaling activity and no apparent irregularity in the amount of glycosylation, indicate a benign nature. However, a deeper analysis using mass spectrometry might reveal subtle changes in the glycosylations or other post-translation modifications that are below the resolution of the WB analysis after PNGaseF treatment.

Furthermore, our assays cannot detect splicing effects. Indeed, in silico analyses predicted the creation of a novel splice site for *JAG1* c.1345A>G p.(Ile449Val) (SpliceAI prediction score for donor gain: 1), detected in patient 3 with clinical ALGS (Table S3) making a splicing defect the most likely effect of the variant. Since our culture model exclusively employs expression vectors containing cloned cDNA, which lack introns, the splicing effect cannot be tested using these methods. We can therefore conclude that our functional analyses enable reclassification of a VUS as likely pathogenic if a pathogenic effect is detected, but it does not allow a VUS to be classified as likely benign if our assays show no pathogenic effect.

However, at least the NOTCH2 variant p.(His2032Asn) could be readily reclassified as likely benign based on the recently published version of gnomAD population data (minor allele frequency in subpopulation: 0.2%, 3 homozygotes, version 4.1.0) (Table S4). Especially for one of the two carriers of this variant, patient 8 with a clinically classic Alagille syndrome, the presence of another previously unknown variant in *JAG1* or *NOTCH2* is highly likely. However, since it has not yet been found in either of the two patients, not even by subsequent short-read genome sequencing an epigenetic mechanism might be involved. For all other patients in this study, the genetic diagnosis was based on exome sequencing, which does not cover large parts of the non-coding regions, particularly *JAG1* and *NOTCH2*, which are composed of many large introns.

Alagille syndrome has primarily been proposed to be a result of haploinsufficiency (Oda et al. 1997). Some studies also suggest a dominant negative mechanism for some *JAG1* missense variants and variants that lead to premature termination codons (PTC), but do not underlie nonsense-mediated decay (NMD). Those variants can result in soluble forms of JAG1, potentially able to compete with the membrane bound form of the ligand and possibly antagonize Notch signaling (Boyer-Di Ponio et al. 2007). Our data do not suggest that any of our variants show a dominant-negative effect. However, testing clearly dominant-negative control variants would be needed to assess if the assay is sensitive enough to detect these effects. Furthermore, our assay is unable to discriminate between PTC-producing variants that underlie NMD and those that do not. For the initiation of NMD according to the canonical NMD model, exon junction complexes, which were introduced during splicing, are necessary (Le Hir et al. 2001; Thermann et al. 1998). As we use (intron-less) cDNA-based expression vectors, NMD cannot take place in our in vitro assay, even if it would do so in vivo.

Nevertheless, the HEK293T-LNA cell line we generated still outperforms the WT cell lines in providing conditions for more sensitive and targeted variant analysis of Notch pathway components. While subtle, biologically relevant effects may remain undetectable in the context of an overexpression system, the LNA system utility is particularly evident for JAG1 variants, where the dynamic range of activity is relatively narrow, but it also improves the robustness of NOTCH2 assays by enabling “cleaner” receptor–ligand interactions. This might prove to be beneficial also in other Notch-driven disease context, such as NOTCH2-driven Hajdu-Cheney disease, NOTCH1-driven Adams-Oliver syndrome, or NOTCH3-driven infantile myofibromatosis, where the NOTCH1/2/3 parental line for our LNA cells was used for the first time (Mašek and Andersson 2017; Wu et al. 2021).

In summary, our analyses, especially by using the HEK293T-LNA cell line, allow for a more sensitive comprehension of the *JAG1* and *NOTCH2* variants impact on the Notch signaling pathway activity and, thus, an improved interpretation and classification of variants involved in ALGS, as demonstrated on NOTCH2 variant p.(Cys412Phe).

## Electronic Supplementary Material

Below is the link to the electronic supplementary material.

Supplementary Methods and Results

## Data Availability

Patient and variant information can be found at ClinVar (SCV007100086-SCV007100093). Further data are available from the corresponding authors on reasonable request.
