# Supplementary material for: Improved functional JAG1 and NOTCH2 variant testing in patients with clinical or suspected Alagille syndrome using new low-Notch activity cells
Source: Hum Genet. 2026 Apr 18;145(1):39. doi: 10.1007/s00439-026-02832-7 (PMC13090200; doi:10.1007/s00439-026-02832-7)
Supplement: Supplementary file 1 — Supplementary Methods and Results [file 439_2026_2832_MOESM1_ESM.docx]

**Improved functional *JAG1* and *NOTCH2* variant testing in patients with clinical or suspected Alagille syndrome using new Low-Notch Activity cells**

Buhl, Nicole^1^; Pfister, Eva-Doreen^1^; Oliveira, Daniel V.^2,3^; Turetti, Fabio^2^; Lurz, Eberhard^4^; Baumann, Ulrich^1^; Di Donato, Nataliya^5^; Illig, Thomas^6^; Skawran, Britta^5^; Andersson, Emma R.^7^; Mašek, Jan^2,3^*; Stalke, Amelie^5^*

^1^Pediatric Gastroenterology and Hepatology, Hannover Medical School, Hannover, Germany

^2^Department of Cell Biology, Faculty of Science, Charles University, Prague, Czech Republic

^3^Institute of Organic Chemistry and Biochemistry, Czech Academy of Sciences, Prague, Czech Republic

^4^Department of Pediatrics, Dr. von Hauner Children’s Hospital, University Hospital, LMU Munich, Munich, Germany

^5^Department of Human Genetics, Hannover Medical School, Hannover, Germany

^6^Hannover Unified Bank, Hannover Medical School, Hannover, Germany

^7^Department of Cell and Molecular Biology, Karolinska Institute, Stockholm, Sweden

*Corresponding authors

**Corresponding authors:**

Amelie Stalke, Department of Human Genetics, Hannover Medical School, Carl-Neuberg-Str.1, 30625 Hannover, Germany. phone: +49 511/53282517, fax: +49511/5324521, email: Stalke.Amelie@mh-hannover.de

Jan Mašek, Department of Cell Biology, Charles University, Faculty of Science, [Viničná 7](https://www.google.com/maps/search/Vini%C4%8Dn%C3%A1+7?entry=gmail&source=g), 12800 Prague, Czech Republic, phone: |+420 221 951 624, email: jan.masek@natur.cuni.cz

Supplementary Methods

HEK293T Low Notch Activity (HEK293T-LNA) cell generation by CRISPR/Cas9 mutagenesis and Cell culture of HepG2, Huh7 and HEK293T wild-type cell lines

Human embryonic kidney cells HEK293T (kindly provided by Johann Meyer, Institute of Experimental Hematology, MHH, Hannover, Germany) were cultured in Dulbecco's Modified Eagle Medium (DMEM, Merck, #D5796), 10 % fetal calf serum (FCS, Merck, #S0615), 1 % sodium pyruvate (Merck, #S8636) and 100 U/mL penicillin/streptomycin (sigma, #P0781). Human liver carcinoma cell lines HepG2 and Huh7 (kindly provided by Professor Nam-Ho Huh, Department of Cell Biology, Graduate School of Medicine, Dentistry and Pharmaceutical Sciences, Okayama University, Okayama, Japan) were cultured in DMEM (PAN-Biotech, #P04-05550) containing 10 % FCS and 100 U/mL penicillin/streptomycin.

We used the NOTCH1/2/3 KO HEK293T (1) (Kindly provided by Urban Lendahl, Karolinska Institute, Sweden) to develop a new HEK293T cell line, depleted of NOTCH1/2/3 receptors and multiple Notch ligands, and thus largely devoid of Notch signaling activity – Low Notch Activity HEK293T cells (HEK293T-LNA). We achieved this by using the CRISPR/Cas9 px459 system (AddGene #62988) (2), performing multiple rounds of sgRNA/Cas9 transfection and selection, followed by limiting dilution and clonal expansion. The clones arising from individual cells were tested by sequencing (3) and Western blotting.

Resulting HEK293T-LNA cells are *NOTCH1/2/3-/-, JAG1/2-/-, DLL1-/in frame del, DLL3-/in frame del, DLL4+/—* (Fig. S1A-C). JAG1-T2A-EGFP expressing HEK293T-LNA cells originate from HEK293T-LNA cells with random genomic integration of the transiently transfected JAG1-T2A-EGFP plasmid that were purified by FACS. Overexpression of the JAG1 was confirmed by Western blotting (Fig. S1D). Both HEK293T-LNA and JAG1-T2A-EGFP HEK293T-LNA cells were cultured in DMEM (High glucose, Glutamax supplement, pyruvate, Thermo Fisher #31966047), supplemented with 10 % fetal bovine serum (FBS, Gibco™, #A5256701), and 1 % of penicilin/streptomycin (Gibco™, #15140122). All cell lines were cultivated at 37 °C and 5 % CO_2_ in a humidified incubator.

Vector constructs

For reporter activity assays pcDNA3_*JAG1* or pcDNA3.1_*JAG1* and pcDNA3.1_*NOTCH2* expression vectors containing the coding region of the *JAG1* (NM_000214.3) and *NOTCH2* (NM_024408.4) gene, respectively, were purchased from Genescript in pcDNA3.1 backbone. For qPCR pcDNA3.1_*NOTCH4* containing the coding region of *NOTCH4* (NM_004557.4) was purchased from Genescript. The JAG1-EGFP vector was subsequently modified using EcoRI and NotI sites to contain a self-cleaving T2A peptide between the JAG1 and EGFP, resulting in JAG1-T2A-EGFP in pcDNA3.1.

For CRISPR/Cas9 mutagenesis px459 vector (AddGene #62988) was used. The used gRNA target sites were selected with CHOP CHOP tool (4-6) (Fig. S1E).

All *NOTCH2* and *JAG1* variants were generated using QuickChange Lightning Site-Directed Mutagenesis Kit (Agilent Technologies, #210518) according to the manufacturer’s protocol. For primer sequences, see Table 1. All sequences were verified by Sanger sequencing either for the insert after cloning into a new backbone or by Full PlasmidSeq (microsynth SeqLab, Göttingen, Germany). As a reporter plasmid Ga981-6 with 12 CSL binding sites upstream of the firefly luciferase open reading frame (12x CSL in Ga50-7) was used (Kind gift by Urban Lendahl).

Table S1: Primer sequences for site directed mutagenesis

| JAG1_ c.3467T>C p.(Val1156Ala) | Forward: 5'GACACACAATTCTGAAGCAGAAGAGGACGACATGG3'  Reverse: 5'CCATGTCGTCCTCTTCTGCTTCAGAATTGTGTGTC3' |
| --- | --- |
| JAG1_ c.53T>G p.(Leu18Arg) | Forward: 5'CTAAGCCTCCTGCGCGCCCTGCTCTGTG3'  Reverse: 5'CACAGAGCAGGGCGCGCAGGAGGCTTAG3' |
| JAG1_ c.316A>G p.(Thr106Ala) | Forward: 5'CATCGGGGGCAACGCCTTCAACCTCAAG3'  Reverse: 5'CTTGAGGTTGAAGGCGTTGCCCCCGATG3' |
| JAG1_ c.1912T>C p.(Cys638Arg) | Forward: 5'GTGAGAGCAACCCTCGTAGAAACGGTGGC3'  Reverse: 5'GCCACCGTTTCTACGAGGGTTGCTCTCAC3' |
| JAG1_ c.1345A>G p.(Ile449Val) | Forward: 5'GGATGGGTCAGAATTGTGACGTAAATATTAATGACTGCCTTG3'  Reverse: 5'CAAGGCAGTCATTAATATTTACGTCACAATTCTGACCCATCC3' |
| JAG1_ c.551G>A p.(Arg184His) | Forward: 5'CACTTTGAGTATCAGATCCACGTGACCTGTGATGACTAC3'  Reverse: 5'GTAGTCATCACAGGTCACGTGGATCTGATACTCAAAGTG3' |
| JAG1_ c.703C>T p.(Arg235*) | Forward: 5'GTAACAGAGCTATTTGCTGACAAGGCTGCAGTCC3'  Reverse: 5'GGACTGCAGCCTTGTCAGCAAATAGCTCTGTTAC3' |
| JAG1_ c.2101A>C p.(Thr701Pro) | Forward: 5'GGTGGAAAGGAAAGCCCTGCCACTCACGTG3'  Reverse: 5'CACGTGAGTGGCAGGGCTTTCCTTTCCACC3' |
| NOTCH2_c.3995G>A p.(Arg1332His) | Forward: 5'CTGATGGTTTCATTTGCCATTGTCCCCCGGGATTTTC3'  Reverse: 5'GAAAATCCCGGGGGACAATGGCAAATGAAACCATCAG3' |
| NOTCH2_c.6094C>A p.(His2032Asn) | Forward: 5'CAGCCAAGATCCTGTTAGACAATTTTGCCAATCGAGACATC3'  Reverse: 5'GATGTCTCGATTGGCAAAATTGTCTAACAGGATCTTGGCTG3' |
| NOTCH2_c.1235G>T p.(Cys412Phe) | Forward: 5'CTACAAAGGGGCTGACTTCACAGAAGATGTGGATG3'  Reverse: 5'CATCCACATCTTCTGTGAAGTCAGCCCCTTTGTAG3' |
| NOTCH2_c.1331G>A p.(Cys444Tyr) | Forward: 5'GCCTTCCACTGTGAGTATCTGAAGGGTTATGCAG3'  Reverse: 5'CTGCATAACCCTTCAGATACTCACAGTGGAAGGC3' |
| NOTCH2_c.6007C>T p.(Arg2003*) | Forward: 5'GAAAAATGGGGCCAACTGAGACATGCAGGACAAC3'  Reverse: 5'GTTGTCCTGCATGTCTCAGTTGGCCCCATTTTTC3' |
| NOTCH2_c.3980A>G p.(Asp1327Gly) | Forward: 5' CCAGTAACATGCCTGGTGGTTTCATTTGCCG 3' Reverse: 5' CGGCAAATGAAACCACCAGGCATGTTACTGG 3' |

Variant nomenclature refers to NCBI transcripts *JAG1* (NM_000214.3) and *NOTCH2* (NM_024408.4)

Transfection

To overexpress JAG1, NOTCH2, or its mutants for western blot, 325000 HEK293T, 500000 Huh7 or 550000 HepG2 cells were seeded per well 24 h before transfection in a 6-well format. For HEK293T or Huh7 transfection 300 ng of the respective pcDNA3/.1 vector, 10 µl Lipofectamine 2000 (Thermo Fisher, #11668019) and 1700 ng MIGR1 (kindly provided by Warren Pear via Addgene) were used per well. For HepG2 transfection 2500 ng of the respective pcDNA3.1 vector, 3.75 µl Lipofectamine 3000 (Thermo Fisher, #L3000008) and 5 µl P3000 were used per well. The empty vector served as negative control. Medium was changed after 24 h. 48 h after transfection whole cell lysate was isolated.

Western blots in Huh7 and HEK293T wild-type cell lines:

Harvested cells were lysed in RIPA buffer (Sigma Aldrich, #R0278) containing protease and phosphatase inhibitor (Thermo Scientific™, #A32961). The influence of *JAG1* variants on the glycosylation of proteins was analyzed by digestion with Peptide-N-glycosidase F (PNGase F) (New England Biolabs, #P0704S). For this purpose, 1-20 µg protein was mixed with 10x Glycoprotein Denaturing Buffer and filled up to 10 µL with H_2_O. After denaturing for ten minutes at 100°C, cooling on ice and centrifuging for 10 sec, the samples were mixed with 2 µL GlycoBuffer 2 (10x), 2 µL 10 % NP-40 and 6 µL H_2_O. After the addition of 1 µL PNGase F, the samples were incubated for 1 h at 37°C.

Proteins were separated on a 7.5 %/10 % (JAG1/NOTCH2) sodium dodecyl sulfate polyacrylamide gel (TGX Stain-Free Fast Cast Acrylamide, Biorad, #1610181 and #1610183), and transferred onto an Amersham Protran nitrocellulose membrane (Sigma/Merck, Darmstadt, Germany). The membrane was blocked for 1 h in 5 % nonfat dry milk in TBS-T at room temperature. After incubation with primary rabbit-anti JAG1 (Cell Signaling, #70109S, 1:1000) or rabbit-anti NOTCH2 antibody (Cell Signaling, #5732S, 1:3000) over night at 4 °C and secondary antibody (Goat-anti rabbit IgG-HRP, Abcam, #ab6721, 1:2000/1:10000 for JAG1/NOTCH2, respectively) for 1 h at room temperature, the membrane was washed 3x with TBS-T. The membrane was developed with SuperSignal West Femto Maximum Sensitivity Substrate (Thermo Fisher, #34094). GAPDH (Cell Signaling, #2118S) was used as the loading control.

Western blots in HEK293T-LNA cell line:

Harvested cell were lysed in RIPA Lysis Buffer (25mM TRIS-HCl pH 7.6, 150mM NaCl, 1% NP-40, 1% Sodium Deoxycholate, 0.1% SDS) containing the Protease and phosphatase inhibitor (Thermo Scientific, #A32961). The protein concentrations were determined with the Pierce™ BCA Protein Assay Kit (Thermo Scientific, #23225) using bovine serum albumin as standard. For western blotting, aliquots of 20 μg were denatured by boiling in Laemmli Sample Buffer (4x), separated by SDS-PAGE gel 8%, and transferred onto PVDF membranes (Amersham™ Hybond® P Western blotting membranes, PVDF, #10600021) by electroblotting.

The membrane was blocked in 1X TBST (Tris-buffered saline, 0.1% Tween 20), 5% dry milk for 1h at room temperature followed by overnight incubation at 4°C with rabbit-anti NOTCH1 (Cell Signaling, #3608S, 1:1000), rabbit-anti NOTCH2 (Cell Signaling, #5732S, 1:1000), rabbit-anti NOTCH3 (Cell Signaling, #5276S, 1:1000), rabbit-anti JAG1 (Cell Signaling, #70109S, 1:1000), rabbit-anti JAG2 (Cell Signaling, #2210S, 1:1000), mouse-anti DLL1 (Santa Cruz, #sc-377310, 1:1000), mouse-anti DLL3 (Sigma-Aldrich, #MABT189, 1:1000), mouse-anti DLL4 (R&D Systems, #AF1389, 1:1000) or rabbit-anti HES1 antibody (Abcam, #ab71559, 1:1000).

The next day membranes were washed 4 times with 1X TBST and incubated with anti-rabbit-HRP (Abcam, #ab205718), anti-mouse-HRP (Abcam, #ab205719) conjugated secondary antibodies at 1:20000 for 1h at room temperature followed by 4 washed with 1X TBST. Membranes were activated for band detection with Pierce™ ECL Western Blotting Substrate (Thermo Fisher Scientific, # 32209). Mouse anti ß-actin (Santa Cruz, sc-47778, 1:1000) was used as loading control.

A summary of all antibodies used in this study is shown in Tab S2.

**Table S2: Antibodies used in this study**

| Antibody | **Catalog number** | **Source** | **Dilution for WB** |
| --- | --- | --- | --- |
| α-NOTCH1 (D1E11) | 3608S | Cell Signaling | 1:1000 |
| α-NOTCH2 (D76A6) | 5732S | Cell Signaling | 1:1000 and 1:3000 |
| α-NOTCH3 (D11B8) | 5276S | Cell Signaling | 1:1000 |
| α-JAG1 (28H8) | 2620S | Cell Signaling | 1:1000 |
| α-JAG1 (D4Y1R) | 70109S | Cell Signaling | 1:1000 |
| α-JAG2 (C23D2) | 2210S | Cell Signaling | 1:1000 |
| α-DLL1 | sc-377310 | Santa Cruz | 1:1000 |
| α-DLL3 | MABT189 | Sigma-Aldrich | 1:1000 |
| α-DLL4 | AF1389 | R&D Systems | 1:1000 |
| α-β-Actin | sc-47778 | Santa Cruz | 1:1000 |
| α-HES1 | ab71559 | Abcam | 1:1000 |
| α-GAPDH (14C10) | 2118S | Cell Signaling | 1:1000 |
| α-Rabbit IgG H&L (HRP) | ab6721 | Abcam | 1:2000 and 1:10000 |
| α-Rabbit IgG H&L (HRP) | ab205718 | Abcam | 1:20000 |
| α-mouse IgG H&L (HRP) | ab205719 | Abcam | 1:20000 |

Luciferase-based Notch signaling assay of NOTCH2 variants with HepG2 and Huh7 cells

Luciferase reporter assay started with seeding 20000 Huh7 or HepG2 cells, free of mycoplasma, per well in white 96 well plates using 100 µl of the corresponding medium without antibiotics in duplicates. The ratio of signal-sending cells (JAG1) and signal-receiving cells (NOTCH2) was 1:1. Transfection was performed 24 h later with 0.5 µl Lipofectamine 2000 (Thermo Fisher, #11668019, for Huh7) or 0.15 µl Lipofectamine 3000 and 0.1 µl P3000 (Thermo Fisher, #L3000008, for HepG2) per well.

The signal-receiving cells were transfected with 40 ng 12xCSL reporter construct, 6 ng (Huh7) or 3 ng (HepG2) of pGL4.70 vector (Promega, #E6881) with an EF1α promoter inserted at the XhoI restriction site upstream of Renilla luciferase (used for normalization) and 50 ng of the respective pcDNA3.1_NOTCH2 vector or the empty vector control.

The signal-sending cells were transfected with 50 ng of the pcDNA3.1_JAG1_WT or empty vector control. 6 h after transfection, the signal-sending cells were washed once with pre-warmed PBS and incubated with 30 μL TrypLE (Thermo Fisher, #12563029) for 5/7 min (Huh7/HepG2) at 37 °C for detachment. The reaction was stopped by adding 120 µl medium without antibiotics, and the cells were detached and separated by repeated pipetting up and down and transferred to the signal-receiving cells. 48 h after transfection, the medium was removed and replaced with 45 μL of fresh medium without antibiotics and luciferase activity was measured with the DualGlo Luciferase Assay System (Promega, #E2920).

Luciferase-based Notch signaling assay of NOTCH2 and JAG1 variants with HEK293T-LNA cells

HEK293T-LNA cells, free of mycoplasma, were seeded in a 24-well plate at a density of 1.25 × 10⁵ cells per well. After 24 h, cells were transfected overnight with 200 ng of 12xCSL-luc reporter and 8 ng of Renilla luciferase plasmids, along with 100 ng of either NOTCH2, NOTCH2 variant of interest, or empty pcDNA3.1 plasmids. The total DNA per well was adjusted to 1 µg by supplementing with pBluescript KSII plasmid (Kind gift from Zbynek Kozmik).

Transfections were performed using Lipofectamine 2000 (Thermo Fisher #11668019), following the manufacturer’s protocol. The following morning, 10000 transfected HEK293T-LNA cells (signal receiver cells) from each condition were co-cultured with 40000 HEK293T-LNA cells stably expressing JAG1 (signal sending cells) in a 96-well plate and incubated overnight. The next day (40 h post-transfection), the medium was replaced, and luciferase activity was measured using the Dual-Glo Luciferase Assay System (Promega, #E2940), according to the manufacturer’s instructions.

For JAG1 variant analysis, HEK293T-LNA cells were transfected with 100 ng of NOTCH2, 200 ng of 12XCSL-luc reporter, and 8 ng of Renilla plasmids. Additional HEK293T-LNA cells were transfected with 100 ng of JAG1, JAG1 variant of interest, or empty pcDNA3.1 plasmids.

Total DNA was again adjusted to 1 µg with pBluescript KSII plasmid, and transfections were performed as described. The following day, 40000 HEK293T-LNA cells transfected with JAG1 vectors (signal sending cells) were co-cultured with 10000 HEK293T-LNA cells transfected with NOTCH2, 12XCSL-luc, and Renilla plasmids (signal receiver cells) in a 96-well plate. After overnight incubation, the medium was replaced, and luciferase activity was measured using the Dual-Glo Luciferase Assay System as described previously.

Sequencing Approach

DNA was extracted from whole blood samples. For whole-exome sequencing, DNA enrichment and library preparation were performed using the xGen Exome Research Panel (Integrated DNA Technologies, Inc., Coralville) or TruSeq Exome (Illumina, San Diego, CA). Sequencing was performed on a NextSeq 500/550 using the NextSeq 500/550 High Output v2 kit (Illumina) or on an MGI DNB SEQ-G400RS using the DNBSEQ-G400RS High-throughput Sequencing Set (BGI, Shenzhen, China). Alignment was performed to the Genome Reference Consortium Human Build 38 (GRCh38) using megSAP, version megSAP-2023_11-315-gc8f66b47 (<https://github.com/imgag/megSAP>).

Variant prioritization and visualization were performed with GSvar, version ngs-bits-2024_08-35-g5b45dc95 (https://github.com/imgag/ngs-bits), Integrative Genomics Viewer (IGV) (PMID: 21221095) version 2.16.0 and with Alamut® visual Plus, version 1.8.1 (Interactive Biosoftware, Rouen, France). One retrospectively evaluated case (patient 3) was referred to us with previously externally performed genetic analyses of the *JAG1* gene by single strand conformation polymorphism analysis and cycle sequencing. Further genetic analyses were impossible as this patient was lost to follow-up.

For all mentioned variants nucleotide numbering starts with the A of the ATG translation initiation site as nucleotide 1. Variant nomenclature refers to NCBI transcripts *JAG1* (NM_000214.3) and *NOTCH2* (NM_024408.4)

qPCR

HEK293T and HEK293T-LNA cells (2.5 x 10^5^ cells/well) were seeded in 24-well plates. For transfection, 2.0 x 10^5^ cells/well were seeded and transfected 16h later with 500ng of pcDNA3.1-hNOTCH4-C_HA plasmid or empty pcDNA3.1. After 24 h, cells were harvested in 1 mL cold TRIzol™ (Thermo Fisher Scientific, #15596026). Total RNA was isolated, and 1µg was treated with DNase I (Thermo Fisher Scientific, #EN0521) for 30 min at 37°C, followed by 1µl of 50mM EDTA for 10 min at 65°C.

cDNA was synthesized using the RevertAid transcriptase (Thermo Fisher Scientific, # EP0442) and subsequently diluted with 80µ of nuclease-free water. qPCR was performed using 2µl of cDNA and Luna® Universal qPCR Master Mix (NEB, #M3003S) on a LightCycler® 480 instrument. Cycling conditions: 42 cycles with a 60°C annealing temperature. Primers used:

| **Gene** | **Sequence 5’-3’** |
| --- | --- |
| *GAPDH_*F | GCTCTCTGCTCCTCCTGTTC |
| *GAPDH_*R | ACGACCAAATCCGTTGACTC |
| *UBC_*F | TGACCAGCAGAGGTTGATCTT |
| *UBC_*R | TTTGCTTTGACGTTCTCGATAG |
| *NOTCH1_*F | GAGGCGTGGCAGACTATGC |
| *NOTCH1_*R | CTTGTACTCCGTCAGCGTGA |
| *NOTCH2_*F | CCTTCCACTGTGAGTGTCTGA |
| *NOTCH2_*R | AGGTAGCATCATTCTGGCAGG |
| *NOTCH3_*F | CGTGGCTACACTGGACCTC |
| *NOTCH3_*R | AGATACAGGTGAACTGGCCTAT |
| *NOTCH4_*F | CCTGGCTCCTTCAACTGCC |
| *NOTCH4_*R | GCAAGTAGGTCCAGACAGGT |
| *NOTCH4-*cDNA_F | TTCCACTGTCCTCCTGCCAGAA |
| *NOTCH4-*cDNA_R | TGGCACAGGCTGCCTTGGAATC |

Ct values of the qPCR assay can be found here: <https://www.ebi.ac.uk/biostudies/studies/S-BSST2221>.

Supplementary Results

| **Patient 6** | 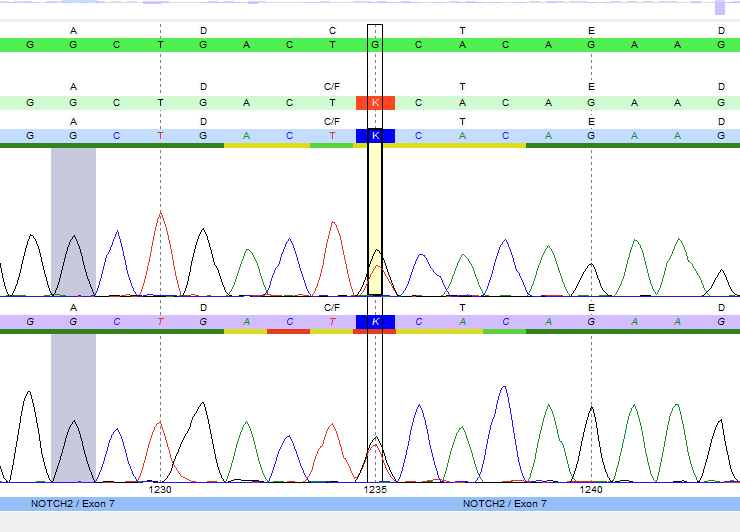 |
| --- | --- |
| **Father of patient 6** | 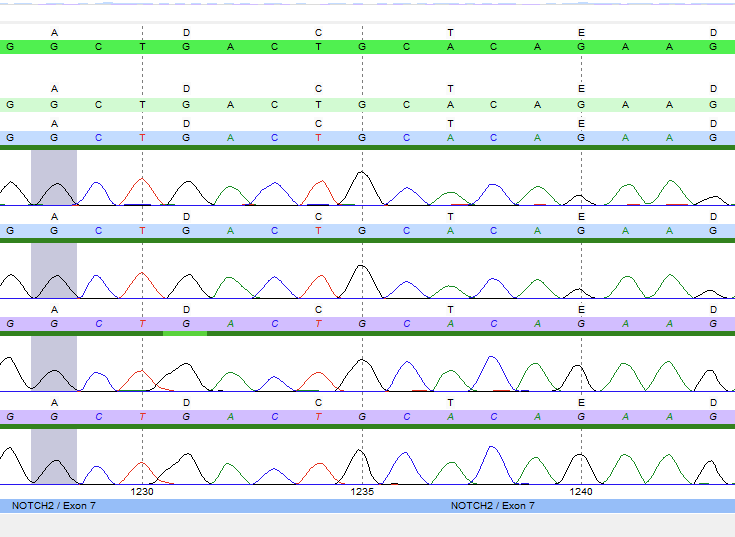 |
| **Mother of patient 6** | 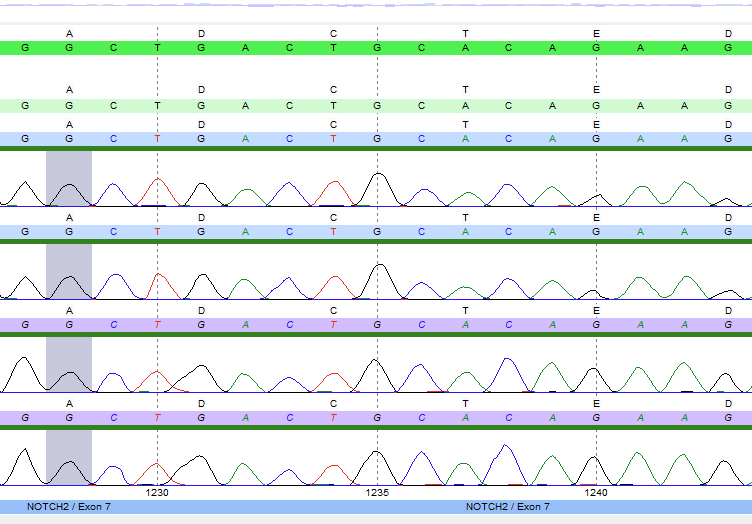 |

**Figure S1: Sanger Sequencing results of DNA from peripheral blood of patient 6 and her parents show *de novo* occurrence of *NOTCH2* variant c.1235G<T p.(Cys412Phe) (NM_024408.4).** Maternity and paternity were not confirmed.


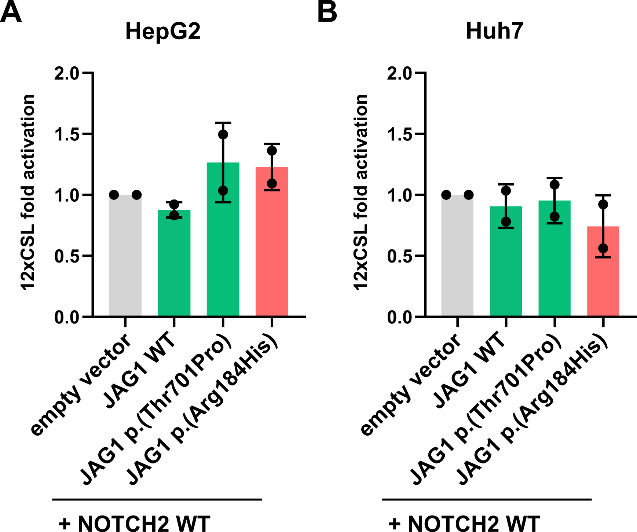


**Figure S2: No detectable influence of different *JAG1* variants on the NOTCH signalling pathway activity in the Huh7 and HepG2 cell lines. (A, B)** co-cultures of (A) WT HepG2 transiently expressing NOTCH2 WT (receiver cells) and WT HepG2 cells transiently expressing different JAG1 variants (sending cells), or (B) WT Huh7 transiently expressing NOTCH2 WT (receiver cells) and WT Huh7 cells transiently expressing different JAG1 variants (sending cells). NICD-induced 12x CSL activation of firefly luciferase was measured and normalized to Renilla luciferase activity. Luciferase activity was normalized to the empty vector control. Biological replicates n = 2. Error bars indicate standard error of the mean (SEM). The Wildtype (WT) and the benign control are shown in green, the pathogenic control is shown in red.


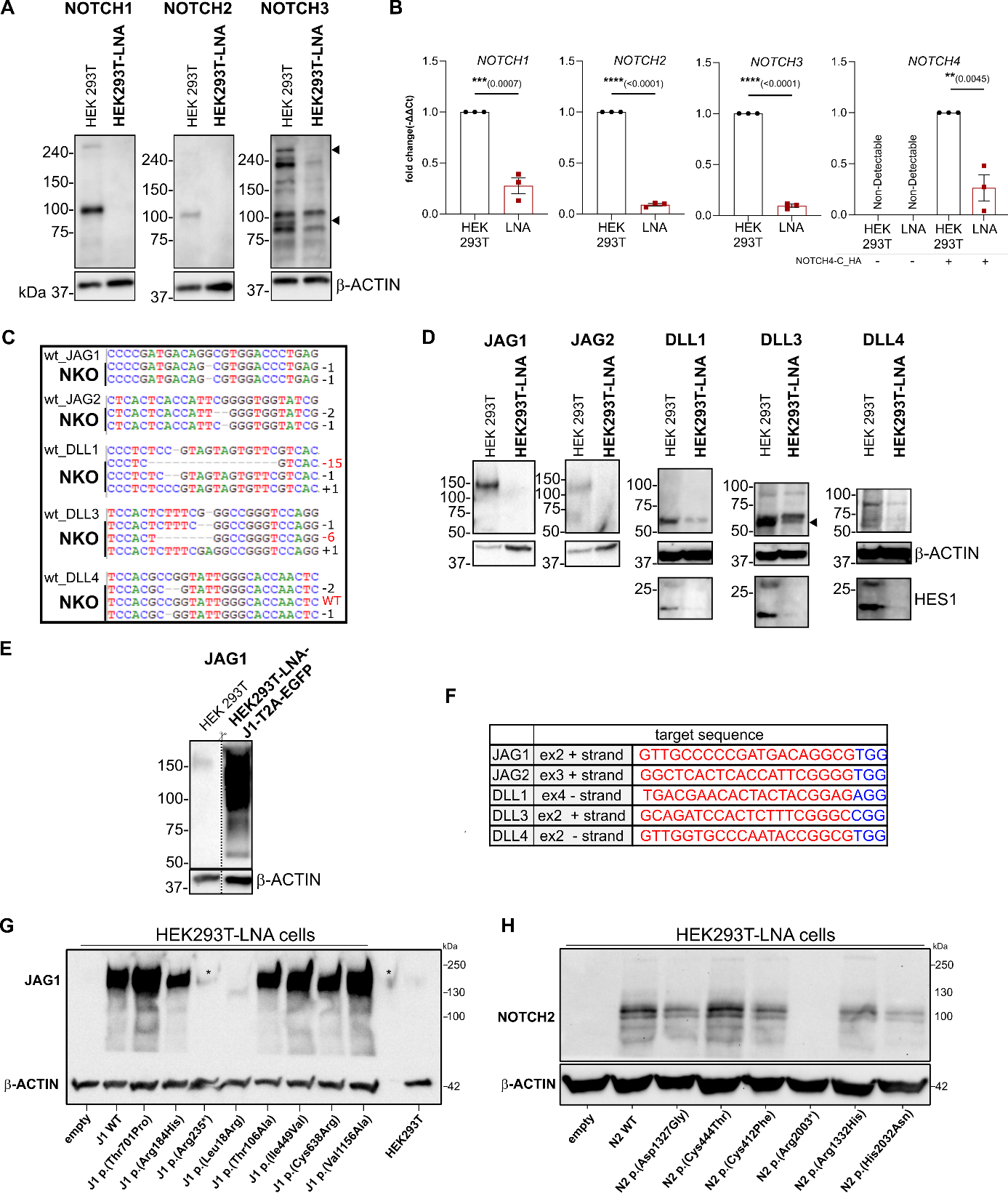


**Figure S3: Validation of newly developed HEK293T-LNA cell line and JAG1 and NOTCH2 variants expression. (A, B)** HEK293T-LNA cell line was validated by Western blot (A) and qRT-PCR (B) for the depletion of NOTCH receptors (NOTCH1/2/3)(A), and expression of *NOTCH1/2/3/4* mRNA (B), in case of *NOTCH4* control transfected with NOTCH4-HA was used to confirm primer functionality. (**C, D**) Sequencing (C), and Western blot (D) were used to confirm the depletion of NOTCH ligands in HEK293T-LNA. Expression of endogenous Notch target gene HES1 (D) was used as a reference of NOTCH pathway inactivity, black arrows indicate reference band. (**E**) Level of JAG1 protein in the newly generated JAG1-T2A-EGFP expressing HEK293T-LNA cells. (**F**) gRNA target sites selected with CHOP CHOP tool (https://chopchop.cbu.uib.no/). (**G, H**) Western blot shows protein levels of JAGGED1 (G) and NOTCH2 (H) variants after transient transfection in HEK293T LNA cells. * indicates an overspill; ß-actin was used as a loading control.


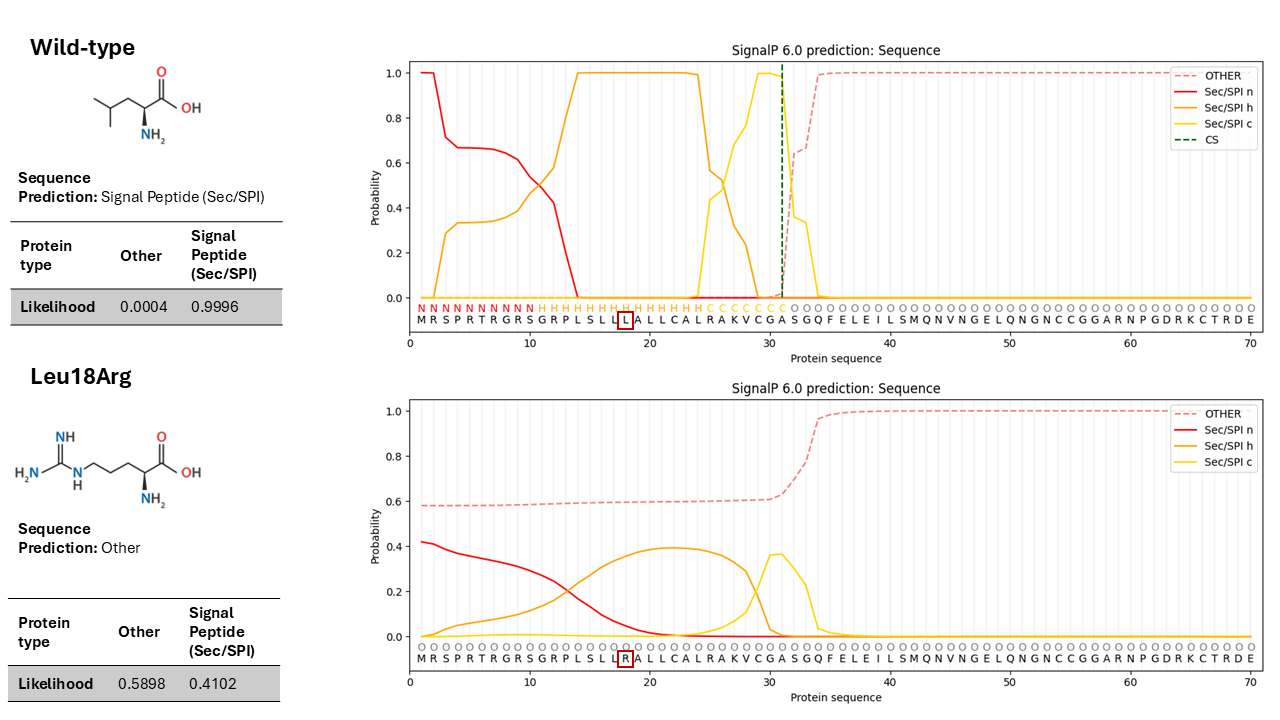


**Figure S4:** **Predicted presence of a signal peptide for JAG1 p.(Leu18Arg) compared to wild-type using SignalP v. 6.0(7).** Changing JAG1 Leu18 to Arg18 results in a notable loss of hydrophobicity The hydrophobic region of signal peptides is critical for recognition by the signal recognition particle (SRP), a ribonucleoprotein complex that binds to the signal peptide of newly synthesized proteins and guides the ribosome–mRNA complex to the endoplasmic reticulum (ER) (8). The impaired interaction between SRP and signal peptide thus likely prevents the protein from entering the ER. This, in turn, leads to protein mislocalization and may activate quality control pathways as “regulation of aberrant protein production” (RAPP) (9-13), leading to low protein levels, as shown in **Fig. 2** and **Fig. S3G**. Plotted are marginal probabilities for signal peptide regions. Sec/SPI: Sec peptide (secretory signal peptides transported by the Sec translocon and cleaved by Signal Peptidase I), *n:* N-terminal region of the signal peptide; *h*: center hydrophobic region of the signal peptide; *c:* c-terminal region of the signal peptide, CS: cleavage site; other: No signal peptide predicted

**Table S3: Characteristics and classification of *JAG1* variants of interest and control *JAG1* variants in this study.**

| **Gene** | ***JAG1* (NC_000020.11, NM_000214.3)** | | | | | | | |
| --- | --- | --- | --- | --- | --- | --- | --- | --- |
| **Variant** | **g.10673478A>C**  **c.53T>G**  **p.(Leu18Arg) het** | **g.10672772T>C**  **c.316A>G**  **p.(Thr106Ala) het** | **g.10649525T>C**  **c.1345A>G**  **p.(Ile449Val) het** | **g.10646058A>G**  **c.1912T>C**  **p.(Cys638Arg) het** | **g.10639688A>G**  **c.3467T>C**  **p.(Val1156Ala) het** | **g.10645368T>G**  **c.2101A>C**  **p.(Thr701Pro)** | **g.10656450G>A c.703C>T**  **p.(Arg235*)** | **g.10658611C>T c.551G>A**  **p.(Arg184His)** |
| **Patient** | **1** | **2** | **3** | **4** | **5** | **Benign control** | **Pathogenic nonsense control** | **Pathogenic missense control** |
| **gnomAD v4.1 (14)** | no entry | AFR: 0.01201 %, Total: 16 het | no entry | no entry | NFE: 0.01830 %, Total: 228 het | ADM: 5 %,  Total: 11906 het | Total:6.197x10^-5^ %,  1 het | no entry |
| **ClinVar (15)** | 1 entries (cl.: 1x LP) VCV002506465.3 | 3 entries (cl.: 2xVUS, 1x B) VCV001445033.11 | 1 entry (cl.: VUS) VCV001361164.5 | no entry | 6 entries (cl.:4xVUS, 2xLB) VCV000424319.42 | 1 entry (cl.: LB)  VCV000803601.1 | 7 entries (cl.: 7xP) VCV000234320.22 | 7 entries (cl.: 6xP, 1x n.p.) VCV000007620.19 |
| **REVEL-Score (16)** | 0.349 | 0.661 | 0.414 | 0.876 | 0.394 | 0.951 | n.a. | 0.949 |
| **Splicing prediction*** | unremarkable | unremarkable | new donor splice site predicted at c.1344, Splice AI: [0-1]: 1; MaxEntScan:  [0-12]:_🡪10.93 | unremarkable | unremarkable | unremarkable | unremarkable | unremarkable |
| **Literature** | (17) | - | - | - | - | - | (18-24) | (17, 18, 20, 23, 25-29) |
| **Inheritance** | unknown | unknown | unknown | unknown | unknown | n.a. | n.a. | n.a. |
| **ACMG criteria** | PS3, PM2_supp, PP2  (6 points) | PP3, PP2  (2 points) | PM2_supp, PP3, PP2  (3 points) | PM2_supp, PP3_mod, PP2  (4 points) | PP2  (1 point) | BA1  (stand alone) | PVS1, PM2_supp, PS4_mod, PM6_supp, (12 points) | PP3_strong, PM2_supp, PS3, PM1_supp, PS4_mod, PP2 (13 points) |
| **Classification^#^** | Likely pathogenic | VUS | VUS | VUS | VUS | benign | pathogenic | pathogenic |
| **According to the research performed in this study** | | | | | | | | |
| **Functional results compared to WT** | JAG1 protein level↓, glycosylation↓, 12xCSL fold activation↓ | JAG1 protein level↓, no difference in glycosylation and 12xCSL fold activation | No difference in JAG1 protein level, glycosylation and 12xCSL fold activation | No difference in JAG1 protein level, glycosylation and 12xCSL fold activation | No difference in JAG1 protein level, glycosylation and 12xCSL fold activation | No difference in JAG1 protein level, glycosylation and 12xCSL fold activation | No JAG1 protein detectable,  no difference in glycosylation,  12xCSL fold activation↓ | JAG1 protein level↓,  no difference in glycosylation,  12xCSL fold activation↓ |
| **ACMG criteria^#^** | PS3, PS4_mod, PM2_supp, PP2  (8 points) | PP3, PP2  (2 points) | PM2_supp, PP3, PP2  (3 points) | PM2_supp, PP3_mod, PP2  (4 points) | PP2  (1 point) | BA1  (stand alone) | PVS1, PM2_supp, PS4_mod, PM6_supp, PS3_supp (13 points) | PP3_strong, PM2_supp, PS3, PM1_supp, PS4_mod, PP2 (13 points) |
| **(Re-) Classification**^#^ | Likely pathogenic | VUS | VUS | VUS | VUS | benign | pathogenic | pathogenic |

*according to Splice AI(30) and MaxEntScan(31); ^#^Variant classification according to the current ACMG Standards and Guidelines using the point system and the ClinGen Variant Classification Guidance (32-34). ACMG: American College of Medical Genetics and Genomics, ADM: Admixed American, AFR: African, B: benign, cl.: classification, n.a.: not applicable, n.p.: not provided, het: heterozygous, LB: likely benign, LP: likely pathogenic, NFE: non-Finish European, P: pathogenic, VUS: variant of uncertain significance, WT: Wild-type

**Table S4: Characteristics and classification of *NOTCH2* variants of interest and control *NOTCH2* variants in this study.**

| **Gene** | ***NOTCH2* (NC_000001.11, NM_024408.4)** | | | | | |
| --- | --- | --- | --- | --- | --- | --- |
| **Variant** | **g.119968106C>A**  **c.1235G>T**  **p.(Cys412Phe) het** | **g.119926509C>T**  **c.3995G>A**  **p.(Arg1332His) het** | **g.119916628G>T**  **c.6094C>A**  **p.(His2032Asn) het** | **g.119926524T>C**  **c.3980A>G p.(Asp1327Gly)** | **g.119917685G>A**  **c.6007C>T**  **p.(Arg2003*)** | **g.119967555C>T**  **c.1331G>A**  **p.(Cys444Tyr)** |
| **Patient** | **6** | **7** | **8, 9** | **Benign control** | **Pathogenic nonsense control** | **Pathogenic missense control** |
| **gnomAD v4.1**  **minor allele frequency** | no entry | AFR: 0.02937 %,  Total: 79 het | NFE: 0.2 %,  Total: 2815 het,  3 hom | SAS: 5 %,  Total:14038 het, 215 hom | Total: 6.20x10^-5^ %, 1 het | No entry |
| **ClinVar** | no entry | 4 entries (cl.: 3x VUS, 1x n.p.) VCV000134973.1 | 9 entries (cl. 2x B, 7x LB) VCV000286650.38 | 7 entries (cl.: 5x B, 1xLB, 1x n.p.) VCV000134972.19 | 4 entries (cl.: 3x P, 1x LP)  VCV000041266.9 | 1 entry (cl.: 1x P)  VCV000009230.2 |
| **REVEL-Score(16)** | 0.915 | 0.231 | 0.335 | 0,401 | n.a. | 0,968 |
| **Splicing prediction*** | unremarkable | unremarkable | unremarkable | unremarkable | unremarkable | unremarkable |
| **Literature** | - | (35) | (35-38) | (39-41) | (23, 42-46) | (23, 39, 42) |
| **Inheritance** | *de novo °* | unknown | Paternal (P8), unknown (P9) | n.a. | n.a. | n.a. |
| **ACMG criteria** | PM2_supp, PP3_mod, PP2, PM6_supp   (5 points) | BP4, PP2  (0 points) | BS1, BS2_supp, PP2 (-4 points) | BA1 (stand alone) | PVS1_strong,  PM2_supp, PS4_mod, PS3_supp,  PM6_supp (9 points) | PP3_strong, PM2_supp, PP2, PS3_supp, PP1_mod (9 points) |
| **Classification^#^** | VUS | VUS | Likely benign | benign | Likely pathogenic | Likely pathogenic |
| **According to the research performed in this study** | | | | | | |
| **Functional results compared to WT** | 12xCSL fold activation↓ | No difference in 12xCSL fold activation | No difference in 12xCSL fold activation | No difference in 12xCSL fold activation | 12xCSL fold activation↓ | 12xCSL fold activation↓ |
| **ACMG criteria** | PM2_supp, PP3_mod, PP2, PM6_supp, PS3_supp  (6 points) | BP4, PP2  (0 points) | BS1, BS2_supp, PP2 (-4 points) | BA1 (stand alone) | PVS1_strong,  PM2_supp, PS4_mod, PS3_supp,  PM6_supp   (9 points) | PP3_strong, PM2_supp, PP2, PS3_supp, PP1_mod   (9 points) |
| **(Re-) Classification^#^** | Likely pathogenic | VUS | Likely benign | benign | Likely pathogenic | Likely pathogenic |

*according to Splice AI(30) and MaxEntScan(31); ^#^Variant classification according to the current ACMG Standards and Guidelines using the point system and the ClinGen Variant Classification Guidance (32-34); ° For segregation analyses results see Figure S1.

ACMG: American College of Medical Genetics and Genomics, AFR: African, B: benign, cl.: classification, n.a.: not applicable, n.p.: not provided, het: heterozygous; LB: likely benign, LP: likely pathogenic, NFE: non-Finish European, P: pathogenic, SAS: South Asian, VUS: variant of uncertain significance, WT: Wild-type

**Supplementary References**

1. Wu D, Wang S, Oliveira DV, Del Gaudio F, Vanlandewijck M, Lebouvier T, et al. The infantile myofibromatosis NOTCH3 L1519P mutation leads to hyperactivated ligand-independent Notch signaling and increased PDGFRB expression. Dis Model Mech. 2021;14(2).

2. Cong L, Ran FA, Cox D, Lin S, Barretto R, Habib N, et al. Multiplex genome engineering using CRISPR/Cas systems. Science. 2013;339(6121):819-23.

3. Bell CC, Magor GW, Gillinder KR, Perkins AC. A high-throughput screening strategy for detecting CRISPR-Cas9 induced mutations using next-generation sequencing. BMC Genomics. 2014;15(1):1002.

4. Labun K, Montague TG, Krause M, Torres Cleuren YN, Tjeldnes H, Valen E. CHOPCHOP v3: expanding the CRISPR web toolbox beyond genome editing. Nucleic acids research. 2019;47(W1):W171-w4.

5. Labun K, Montague TG, Gagnon JA, Thyme SB, Valen E. CHOPCHOP v2: a web tool for the next generation of CRISPR genome engineering. Nucleic acids research. 2016;44(W1):W272-6.

6. Montague TG, Cruz JM, Gagnon JA, Church GM, Valen E. CHOPCHOP: a CRISPR/Cas9 and TALEN web tool for genome editing. Nucleic acids research. 2014;42(Web Server issue):W401-7.

7. Teufel F, Almagro Armenteros JJ, Johansen AR, Gíslason MH, Pihl SI, Tsirigos KD, et al. SignalP 6.0 predicts all five types of signal peptides using protein language models. Nat Biotechnol. 2022;40(7):1023-5.

8. Nilsson I, Lara P, Hessa T, Johnson AE, von Heijne G, Karamyshev AL. The code for directing proteins for translocation across ER membrane: SRP cotranslationally recognizes specific features of a signal sequence. J Mol Biol. 2015;427(6 Pt A):1191-201.

9. Tikhonova EB, Karamysheva ZN, von Heijne G, Karamyshev AL. Silencing of Aberrant Secretory Protein Expression by Disease-Associated Mutations. J Mol Biol. 2019;431(14):2567-80.

10. Pinarbasi ES, Karamyshev AL, Tikhonova EB, Wu IH, Hudson H, Thomas PJ. Pathogenic Signal Sequence Mutations in Progranulin Disrupt SRP Interactions Required for mRNA Stability. Cell reports. 2018;23(10):2844-51.

11. Karamyshev AL, Patrick AE, Karamysheva ZN, Griesemer DS, Hudson H, Tjon-Kon-Sang S, et al. Inefficient SRP interaction with a nascent chain triggers a mRNA quality control pathway. Cell. 2014;156(1-2):146-57.

12. Tikhonova EB, Gutierrez Guarnizo SA, Kellogg MK, Karamyshev A, Dozmorov IM, Karamysheva ZN, et al. Defective Human SRP Induces Protein Quality Control and Triggers Stress Response. J Mol Biol. 2022;434(22):167832.

13. Karamysheva ZN, Karamyshev AL. Aberrant protein targeting activates quality control on the ribosome. Front Cell Dev Biol. 2023;11:1198184.

14. gnomAD - Genome Aggregation Database [Available from: <https://gnomad.broadinstitute.org/>.

15. ClinVar [Available from: <https://www.ncbi.nlm.nih.gov/clinvar/>.

16. Ioannidis NM, Rothstein JH, Pejaver V, Middha S, McDonnell SK, Baheti S, et al. REVEL: An Ensemble Method for Predicting the Pathogenicity of Rare Missense Variants. Am J Hum Genet. 2016;99(4):877-85.

17. Gilbert MA, Keefer-Jacques E, Jadhav T, Antfolk D, Ming Q, Valente N, et al. Functional characterization of 2,832 JAG1 variants supports reclassification for Alagille syndrome and improves guidance for clinical variant interpretation. Am J Hum Genet. 2024;111(8):1656-72.

18. Krantz ID, Colliton RP, Genin A, Rand EB, Li L, Piccoli DA, et al. Spectrum and frequency of jagged1 (JAG1) mutations in Alagille syndrome patients and their families. Am J Hum Genet. 1998;62(6):1361-9.

19. Colliton RP, Bason L, Lu FM, Piccoli DA, Krantz ID, Spinner NB. Mutation analysis of Jagged1 (JAG1) in Alagille syndrome patients. Hum Mutat. 2001;17(2):151-2.

20. Jurkiewicz D, Gliwicz D, Ciara E, Gerfen J, Pelc M, Piekutowska-Abramczuk D, et al. Spectrum of JAG1 gene mutations in Polish patients with Alagille syndrome. J Appl Genet. 2014;55(3):329-36.

21. Li L, Dong J, Wang X, Guo H, Wang H, Zhao J, et al. JAG1 Mutation Spectrum and Origin in Chinese Children with Clinical Features of Alagille Syndrome. PLoS One. 2015;10(6):e0130355.

22. Sangkhathat S, Laochareonsuk W, Maneechay W, Kayasut K, Chiengkriwate P. Variants Associated with Infantile Cholestatic Syndromes Detected in Extrahepatic Biliary Atresia by Whole Exome Studies: A 20-Case Series from Thailand. Journal of pediatric genetics. 2018;7(2):67-73.

23. Gilbert MA, Bauer RC, Rajagopalan R, Grochowski CM, Chao G, McEldrew D, et al. Alagille syndrome mutation update: Comprehensive overview of JAG1 and NOTCH2 mutation frequencies and insight into missense variant classification. Hum Mutat. 2019;40(12):2197-220.

24. Ogawa Y, Yamamoto A, Yamazawa S, Ikemura M, Hirata Y, Inuzuka R. Decreased smooth muscle cells and fibrous thickening of the tunica media in peripheral pulmonary artery stenosis in Alagille syndrome. Cardiovasc Pathol. 2025;74:107677.

25. Lu F, Morrissette JJ, Spinner NB. Conditional JAG1 mutation shows the developing heart is more sensitive than developing liver to JAG1 dosage. Am J Hum Genet. 2003;72(4):1065-70.

26. Morrissette JD, Colliton RP, Spinner NB. Defective intracellular transport and processing of JAG1 missense mutations in Alagille syndrome. Human molecular genetics. 2001;10(4):405-13.

27. Spinner NB, Colliton RP, Crosnier C, Krantz ID, Hadchouel M, Meunier-Rotival M. Jagged1 mutations in alagille syndrome. Hum Mutat. 2001;17(1):18-33.

28. Heritage ML, MacMillan JC, Anderson GJ. DHPLC mutation analysis of Jagged1 (JAG1) reveals six novel mutations in Australian alagille syndrome patients. Hum Mutat. 2002;20(6):481.

29. Cho JM, Oh SH, Kim HJ, Kim JS, Kim KM, Kim GH, et al. Clinical features, outcomes, and genetic analysis in Korean children with Alagille syndrome. Pediatr Int. 2015;57(4):552-7.

30. Jaganathan K, Kyriazopoulou Panagiotopoulou S, McRae JF, Darbandi SF, Knowles D, Li YI, et al. Predicting Splicing from Primary Sequence with Deep Learning. Cell. 2019;176(3):535-48.e24.

31. Yeo G, Burge CB. Maximum entropy modeling of short sequence motifs with applications to RNA splicing signals. J Comput Biol. 2004;11(2-3):377-94.

32. Richards S, Aziz N, Bale S, Bick D, Das S, Gastier-Foster J, et al. Standards and guidelines for the interpretation of sequence variants: a joint consensus recommendation of the American College of Medical Genetics and Genomics and the Association for Molecular Pathology. Genet Med. 2015;17(5):405-24.

33. Tavtigian SV, Harrison SM, Boucher KM, Biesecker LG. Fitting a naturally scaled point system to the ACMG/AMP variant classification guidelines. Hum Mutat. 2020;41(10):1734-7.

34. ClinGen Variant Classification Guidance [Available from: <https://clinicalgenome.org/tools/clingen-variant-classification-guidance/>.

35. Stalke A, Skawran B, Auber B, Illig T, Schlegelberger B, Junge N, et al. Diagnosis of monogenic liver diseases in childhood by next-generation sequencing. Clin Genet. 2018;93(3):665-70.

36. Rego S, Dagan-Rosenfeld O, Zhou W, Sailani MR, Limcaoco P, Colbert E, et al. High-frequency actionable pathogenic exome variants in an average-risk cohort. Cold Spring Harb Mol Case Stud. 2018;4(6).

37. Kanchi KL, Johnson KJ, Lu C, McLellan MD, Leiserson MD, Wendl MC, et al. Integrated analysis of germline and somatic variants in ovarian cancer. Nat Commun. 2014;5:3156.

38. Chapman G, Moreau JLM, I PE, Szot JO, Iyer KR, Shi H, et al. Functional genomics and gene-environment interaction highlight the complexity of congenital heart disease caused by Notch pathway variants. Human molecular genetics. 2020;29(4):566-79.

39. McDaniell R, Warthen DM, Sanchez-Lara PA, Pai A, Krantz ID, Piccoli DA, et al. NOTCH2 mutations cause Alagille syndrome, a heterogeneous disorder of the notch signaling pathway. Am J Hum Genet. 2006;79(1):169-73.

40. Priest JR, Osoegawa K, Mohammed N, Nanda V, Kundu R, Schultz K, et al. De Novo and Rare Variants at Multiple Loci Support the Oligogenic Origins of Atrioventricular Septal Heart Defects. PLoS Genet. 2016;12(4):e1005963.

41. König E, Volpato CB, Motta BM, Blankenburg H, Picard A, Pramstaller P, et al. Exploring digenic inheritance in arrhythmogenic cardiomyopathy. BMC Med Genet. 2017;18(1):145.

42. Kamath BM, Bauer RC, Loomes KM, Chao G, Gerfen J, Hutchinson A, et al. NOTCH2 mutations in Alagille syndrome. J Med Genet. 2012;49(2):138-44.

43. Lin HC, Le Hoang P, Hutchinson A, Chao G, Gerfen J, Loomes KM, et al. Alagille syndrome in a Vietnamese cohort: mutation analysis and assessment of facial features. Am J Med Genet A. 2012;158a(5):1005-13.

44. Alfares A, Alfadhel M, Wani T, Alsahli S, Alluhaydan I, Al Mutairi F, et al. A multicenter clinical exome study in unselected cohorts from a consanguineous population of Saudi Arabia demonstrated a high diagnostic yield. Mol Genet Metab. 2017;121(2):91-5.

45. Li ZD, Abuduxikuer K, Wang L, Hao CZ, Zhang J, Wang MX, et al. Defining pathogenicity of NOTCH2 variants for diagnosis of Alagille syndrome type 2 using a large cohort of patients. Liver Int. 2022;42(8):1836-48.

46. Ferrandino M, Cardiero G, Di Dato F, Cerrato Y, Vitagliano L, Mandato C, et al. Association of Very Rare NOTCH2 Variants with Clinical Features of Alagille Syndrome. Genes (Basel). 2024;15(8).
